# Missed Opportunities to Diagnose Common Variable Immunodeficiency: a Population-Based Case–Control Study Identifying Indicator Diseases for Common Variable Immunodeficiency

**DOI:** 10.1007/s10875-023-01590-9

**Published:** 2023-09-28

**Authors:** Christina Dahl, Inge Petersen, Frederik V. Ilkjær, Lena Westh, Terese L. Katzenstein, Ann-Brit E. Hansen, Thyge L. Nielsen, Carsten S. Larsen, Isik S. Johansen, Line D. Rasmussen

**Affiliations:** 1 Department of Infectious Diseases, Odense University Hospital, J. B. Winsløws Vej 4, Odense, Denmark; 2 Department of Infectious Diseases, Aarhus University Hospital, Palle Juul-Jensens Blvd. 99, Aarhus, Denmark; 3 Department of Infectious Diseases, Rigshospitalet, Copenhagen University Hospital, Esther Moellersvej 6, Copenhagen, Denmark; 4 Department of Infectious Diseases, Hvidovre Hospital, Copenhagen University Hospital, Kettegaard Allé 30, Copenhagen, Denmark; 5 Department of Pulmonary and Infectious Diseases, North Zealand Hospital, Dyrehavevej 29, Hillerod, Denmark; 6 International Center of Immunodeficiency Diseases, Aarhus University Hospital, Palle Juul-Jensens Blvd. 99, Aarhus, Denmark

**Keywords:** CVID, PID, diagnostic delay, indicators, secondary health care

## Abstract

**Purpose:**

Delayed diagnosis of common variable immunodeficiency (CVID) remains a serious problem. We investigated whether some diseases diagnosed during out-patient visits or admission to hospitals could act as indicator conditions for CVID diagnosis.

**Methods:**

In this nested case–control study, we identified 128 cases diagnosed with CVID in Denmark (1999–2013) and 640 age-, gender-, and region-matched controls. We obtained data on diseases diagnosed at hospitals in the five years before CVID diagnosis from The National Hospital Registry. We grouped hospital diagnoses in 33 major disease categories and 210 subcategories. We used conditional logistic regression to calculate the odds ratios (OR) and 95% confidence intervals (CI) to estimate associations between disease exposure and subsequent CVID.

**Results:**

During the five years preceding a CVID diagnosis, cases had four times as many hospital contacts as the controls (*p* < 0.001). A diagnosis in 18 major disease categories showed a significant OR for subsequent diagnosis of CVID. The most substantial association with a subsequent CVID diagnosis was a diagnosis of lower respiratory tract infections (OR: 29.9; 95% CI: 14.2–63.2) and lung diseases (35.1; 15.0–82.5). We observed a similar association when we removed the last year before diagnosis from analysis and overall, in the years < 1, ≥ 1–3, and ≥ 3–5 before diagnosis, although the absolute number of exposures was small. Twenty-eight specific diseases displayed an at least 3-fold risk of subsequent CVID diagnosis.

**Conclusion:**

Targeted screening for antibody deficiency in patients diagnosed with specific diseases associated with CVID may lead to earlier CVID diagnosis and treatment and thereby potentially reduced morbidity and mortality.

**Supplementary Information:**

The online version contains supplementary material available at 10.1007/s10875-023-01590-9.

## Background

Common variable immunodeficiency (CVID) is the most frequent symptomatic primary immunodeficiency with an estimated prevalence of 1:25,000 among adults in Europe [1, 2]. CVID is characterized by primary hypogammaglobinemia and poor vaccine response and/or reduced frequency of isotype switched memory B-cells. [3, 4]. Patients may present with a wide variety of infections, most commonly sinopulmonary infections and non-infectious complications such as granulomatous diseases, autoimmune disorders, and certain malignancies, e.g., lymphomas [5–7]. A correct and timely diagnosis is challenged by the rare occurrence, the heterogenous presentation, and the lack of knowledge of the disease among non-specialists. Several studies have displayed a large diagnostic delay with a median ranging from 3 to 7 years in Europe [2, 8–13]. Delayed CVID diagnosis is a major problem due to the associated increased morbidity and mortality and reduced quality of life [9, 13, 14]. A previous systematic review based on studies from Europe, America, and Asia found an increased prevalence of recurrent infections and non-infectious diseases at diagnosis, compared to what was expected for the general population [15, 16]. However, to our knowledge, no previous study has investigated the risk of disease compared to a control population in the 5 years prior to CVID diagnosis. We hypothesized that CVID patients would have an increased risk of certain conditions leading to hospital admissions and/or visits in the outpatient department (OPD) compared to the general population and that some of these conditions could act as indicator conditions for a CVID diagnosis. In this study, we aimed to investigate the association between diseases diagnosed at out-patient visits or admission to hospitals and subsequent risk of CVID diagnosis to identify indicator conditions for CVID that could lead to targeted screening for hypogammaglobulinemia and hence an earlier CVID diagnosis.

## Method

### Setting

The Danish population was approximately 5.8 million people in the fourth quarter of 2017 whereas the estimated prevalence of CVID in Denmark was about 1:26,000 [2, 17]. Health care in Denmark is tax-funded and every Danish citizen has free access to primary and secondary health care [18].

### Data Source

We used the unique 10-digit personal identification number (PIN) assigned to all individuals in Denmark at birth or upon immigration to track individuals in the following national healthcare registries:

-*The Danish Civil Registration system* (DCRS), established in 1968, is a national registry which stores information on vital status, residency, and migration for all Danish residents [19].

-*The Danish National Patient Register* (DNPR) which contains information on all patients discharged from Danish hospitals since 1977 and data on all OPD visits and patient seen in the emergency room since 1995 [20]. Diagnoses are coded by the attending physician according to the *International Classification of Diseases (ICD)* 10^th^ revision (ICD-10) since 1994.

-*The Danish Adult Common Variable Immunodeficiency Cohort* (DACC), which has been described in detail elsewhere [2], is a nationwide, retrospective hospital-based cohort study of all Danish CVID patients (≥ 15 years old) followed for care in a Danish hospital from 1994 through 2013, verified by journal review.

### Study Design

The study design was a population-based nested case-control study.

### Participants

Cases were defined as CVID patients identified through DACC and were excluded from our study if they 1) had a missing date of CVID diagnosis, 2) were younger than 18 years old at baseline, 3) were diagnosed before 1 January 1999, and 4) had not been living in Denmark in the five years before diagnosis.

Five age- (+/− 90 days), sex-, and region-matched population controls for each case were selected from the DCRS. The controls had to be alive, living in Denmark during the study period, and not diagnosed with CVID before 2014.

The index date was the date of CVID diagnosis for the matched case.

### Exposure

Using data from DNPR, we extracted hospital diagnoses from OPD contacts and hospital admissions for all study subjects in the five-year period prior to index date. We used methods similar to those used in a previous study for HIV patients [21]. Based on diseases associated with and not previously associated with CVID [6, 16], we defined 33 major disease categories and 210 subcategories based on ICD-10 codes (Table S1). We categorized the diseases according to the anatomical location and type of disease. To diagnose CVID, all other causes of hypogammaglobulinemia must be excluded [4, 8]. Since we were unable to ascertain if cancer patients received immunosuppressive treatment before index date, we excluded this disease category.

We included both primary (A-diagnosis) and secondary (B-diagnosis) diagnostic codes. Diagnoses obtained from emergency rooms but not confirmed subsequently were not included. As an association with use of oral antibiotics has already been shown [22] and our main interest was to observe the risk associated with infections leading to admissions, we excluded OPD contacts for the 13 disease categories concerning infectious diseases. OPD contacts for the remaining 20 disease categories were included. For each (sub)category, we only used first-time diagnoses in the total 5-year period and in the years < 1, ≥ 1–3 and ≥ 3–5 years prior to diagnosis.

### Statistics

We used Pearson’s chi-squared test to compare baseline educational level and first-time hospital contact for the representing 33 major disease categories between cases and controls. Mood’s median test was used to compare the median number of hospital contacts between cases and controls. A two-tailed *p*-value ≤ 0.05 was considered statistically significant. For the 33 major disease categories, conditional logistic regression analysis was used to estimate odds ratios (OR) and 95% confidence intervals (CI) for a subsequent CVID diagnosis in the five years prior to index date. Based on the rare disease assumption, these ORs were further used as an estimate of the relative risk of being diagnosed with CVID. In a sensitivity analysis, we removed the last year before diagnosis from the observations (≥ 1–5 years). We further analyzed 1) the risk in three time-periods before the CVID diagnosis (years < 1, ≥ 1–3, and ≥ 3–5) and 2) the risk associated with 210 subcategories during all five years. Only exposures that included a minimum of one case and one control and had at least a total of 10 participants were further analyzed.

We used Stata 17 (StataCorp LLC, Texas, USA) to manage data and perform statistical analyses.

### Ethics

The study was approved by the Danish Data Protection Agency (Journal no. 2012-58-0018 and 2013-41-2409). All data were stored and processed at Statistics Denmark servers, and no personally identifiable data were accessible. Danish national law does not require ethics approval or individual consent for this type of research.

## Results

**Fig. 1:**
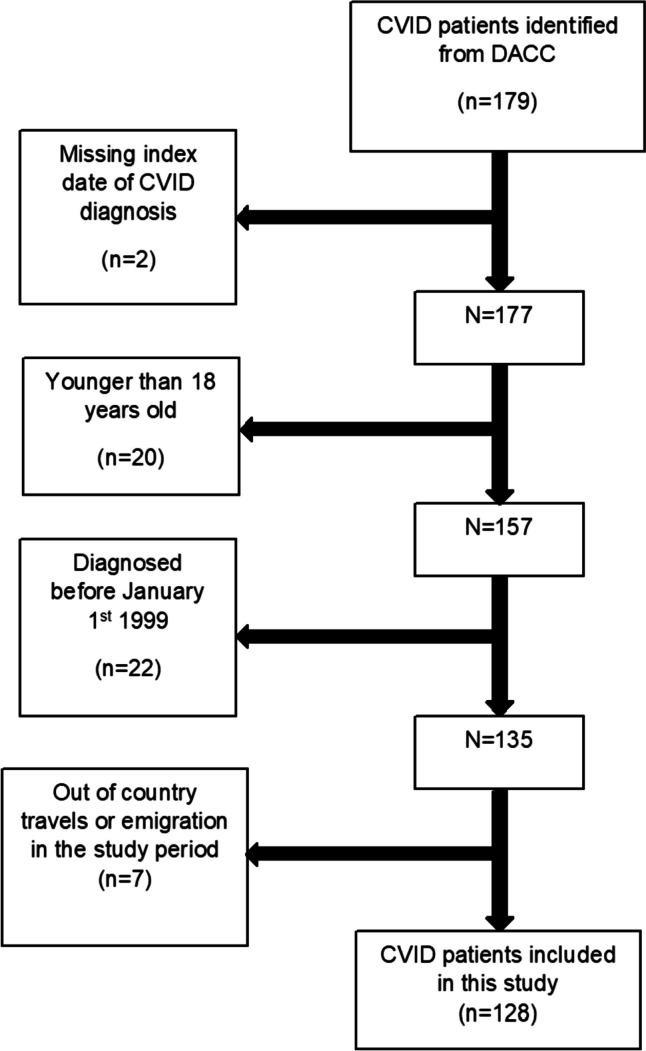
Case sample flowchart. CVID, common variable immunodeficiency; DACC, the Danish Adult Common Variable Immunodeficiency Cohort

We identified 128 cases with CVID from DACC (Fig. 1) and matched them on age, sex, and region with 640 controls. The median age at index date was 47 years (interquartile range; IQR 35–60) and 54% of the sample population were female. There was no significant difference in educational level at baseline (*p*: 0.56). Additional baseline characteristics are shown in Table 1. During the 5 years prior to index date, cases had a total of 2127 hospital contacts (median contacts per person: 12; IQR: 7–19) compared to 3020 contacts among the controls (median contacts per person: 3; IQR: 1–7) (*p* < 0.001). Of these, 116 contacts represented first-time diagnoses (admission or OPD contact) for one of the 33 major diseases categories among cases, and 274 contacts among controls in the five years prior to index date. Among cases 90.6% had at least one hospital contact representing one of the 33 disease categories compared to 42.8% among controls (*p* < 0.001).

**Table 1:** Baseline characteristics of cases and age-, sex-, and region-matched controls

	Cases (*N* = 128)	Controls (*N* = 640)
Sex, *n* (%)		
Male	59 (46)	295 (46)
Female	69 (54)	345 (54)
Calendar year of diagnosis (baseline), *n* (%)		
1999–2003	18 (14.1)	90 (14.1)
2004–2008	39 (30.5)	195 (30.5)
2009–2013	71 (41.4)	355 (41.4)
Age at index date, median (IQR), *n* (%)	47 (35-60)	47 (35-60)
18–39 years	44 (34.4)	220 (34.4)
40–49 years	25 (19.6)	125 (19.6)
50–59 years	26 (20.3)	130 (20.3)
≥60 years	33 (25.8)	165 (25.8)
Symptom duration before diagnosis, *n* (%)		
≤5 years	53 (41.4)	-
5–9 years	13 (10.2)	-
≥10 years	55 (43.0)	-
Unknown	7 (5.4)	-
IgA serum levels, *n* (%)		
≤0.5 g/L	107 (83.6)	-
>0.5–1 g/L	12 (9.4)	-
>1–≥3 g/L	7 (5.4)	-
Unknown	2 (1.6)	-
IgG serum levels, *n* (%)		
0-2 g/L	41 (32.0)	-
>2–3 g/L	27 (21.0)	-
>3–≥5 g/L	46 (36.0)	-
Unknown	14 (11.0)	-
IgM serum levels, *n* (%)		
<0.4 g/L	106 (82.8)	-
>0.4–1 g/L	12 (9.4)	-
>1–≥3 g/L	9 (7.0)	-
Unknown	1 (0.8)	-
Region of Denmark, *n* (%)		
Capital	16 (12.5)	80 (12.5)
Zealand	49 (38.3)	245 (38.3)
Southern Denmark	19 (14.8)	95 (14.8)
Central Jutland	39 (30.5)	195 (30.5)
Northern Jutland	5 (4.0)	25 (4.0)
Educational level, *n* (%)*		
High (>12 years)	32 (25)	177 (27.7)
Medium (12–9 years)	50 (39.1)	265 (41.4)
Low (<9 years) or unknown	46 (35.9)	198 (30.9)

*IQR i*nterquartile range

*P value: 0.56; 95%CI: 0.64–1.24

### Disease Categories in the Five Years Prior to Index Date

Of the 33 major disease categories (for definitions, see Supplementary Information), eighteen categories were associated with an increased risk of subsequent CVID diagnosis (Table 2). A diagnosis of lung diseases was associated with the highest risk (OR: 35.1; 95%CI 15.0–82.5) followed by exposure to lower respiratory tract infections (LRTIs) (29.9; 14.2–63.2). Furthermore, the following infectious categories were associated with an increased risk of a subsequent CVID diagnosis: ear, nose, and throat (ENT) infections (7.5; 2.7–21.1), gastrointestinal infections (7.4; 2.9–21.9), skin infections (7.0; 2.2–22.1), and sepsis (8.8; 2.6–29.9).

Although a diagnosis of most common autoimmune disorders was associated with a trend towards an increased risk, only autoimmune gastrointestinal disorders (7.5; 2.1–26.6) and autoimmune endocrine disorders (5.4; 1.9–15.0) showed a statistically significantly increased risk of subsequent CVID diagnosis. Despite 14 cases being diagnosed with autoimmune hematologic disorders, an OR could not be calculated as no controls had been diagnosed. Finally, concerning the disease category “other diseases” including 15 disease categories, we observed a statistically significant association with a diagnosis for one of the following categories: ENT diseases (2.8; 1.2–6.4), skin diseases (3.8; 1.4–7.5), hematologic diseases (6.5; 2.9–14.8), gastrointestinal diseases (4.6; 2.9–14.8), cardiovascular and non-ischemic heart diseases (non-IHDs) (2.7; 1.6), ischemic heart diseases (IHD) (4.3; 1.7–11.0), rheumatologic diseases (1.8; 1.1–3.0), non-diabetic endocrine diseases (2.8; 1.6–5.1), diabetes type 2 (DM2) and unspecified DM (3.1; 1.3–7.2), and benign tumors (3.6; 1.8–6.9). An OR for diseases associated with the spleen could not be estimated as no controls compared to 6 cases had been diagnosed.

To see if the observed associations were only due to diagnoses given during the last year before index date, we performed a sensitivity analysis in which we removed the last year before diagnosis from the observations and only analyzed the year ≥ 1–5 before index date. Although the OR changed slightly for several categories, no major changes in risks were observed (Table 2).

**Table 2:** Association between hospital contact for 33 disease categories and risk of subsequent CVID diagnosis in the five years prior to a CVID diagnosis

Disease category	5 years Cases	5 years Controls	5 years OR (95%CI)	Sensitivity analysis
				1–5 years OR (95%CI)
Infections				
Ear, nose, and throat infections	9	6	7.5 (2.7–21.1)	6.0 (1.8–19.7)
Eye infections	<3	0	N/A	N/A
Lower respiratory tract infections	49	10	29.9 (14.2–63.2)	32.3 (11.3–92.6)
Gastrointestinal infections	10	7	7.9 (2.9–21.9)	3.6 (1.1–11.3)
CNS infections	<3	0	N/A	N/A
Skin infections	7	5	7.0 (2.2–22.1)	7.5 (2.1–26.6)
STIs and other genital infections	3	7	2.6 (0.6–12.4)	1.8 (0.3–10.2)
Kidney and UTIs	4	6	3.6 (0.9–13.6)	3.0 (0.7–12.6)
Infections of the heart	0	0	N/A	N/A
Joint and bone infections	0	0	N/A	N/A
Sepsis	7	4	8.8 (2.6–29.9)	5.0 (1.3–20.0)
Other viral and fungal infections	<3	3	1.7 (0.2–16.0)	N/A
Other infections	<3	4	1.3 (0.1–11.2)	1.3 (0.1–11.2)
Most common autoimmune disorders				
Autoimmune hematologic disorders	14	0	N/A	N/A
Autoimmune gastrointestinal disorders	6	4	7.5 (2.1–26.6)	5.0 (1.0–24.8)
Autoimmune rheumatic disorders	3	7	2.2 (0.6–9.1)	5.0 (0.7–35.5)
Autoimmune dermatological disorders	3	4	3.8 (0.8–16.8)	7.5 (1.3–44.9)
Autoimmune endocrine disorders	8	4	5.4 (1.9–15.0)	5.0 (1.8–14.3)
Other diseases				
Ear, nose, and throat diseases	11	24	2.8 (1.2–6.4)	2.9 (1.3–6.8)
Eye diseases	9	32	1.5 (0.7–3.3)	1.1 (0.4–3.1)
Lung diseases	46	14	35.1 (15.0–82.5)	28.2 (10.9–72.8)
Neurological diseases	10	25	2.0 (0.9–4.2)	1.3 (0.5–3.2)
Skin diseases	9	15	3.2 (1.4–7.5)	1.8 (0.6–5.7)
Hematological diseases	14	12	6.5 (2.9–14.8)	4.7 (1.8–12.7)
Gastrointestinal diseases	38	60	4.6 (2.8–7.5)	2.8 (1.6-4.8)
Kidney and urinary tract diseases	<3	7	0.7 (0.1–5.8)	1.3 (0.1–11.2)
Spleen diseases	6	0	N/A	N/A
Cardiovascular and non-IHDs	31	75	2.7 (1.6–4.5)	2.9 (1.7–4.9)
Ischemic heart diseases	9	12	4.3 (1.7–11.0)	4.8 (1.7–13.5)
Rheumatological diseases	31	99	1.8 (1.1–3.0)	1.7 (0.98–2.8)
Non-diabetic endocrine diseases	20	40	2.8 (1.6–5.1)	2.5 (1.3–4.9)
DM2 and unspecified DM	9	15	3.1 (1.3–7.2)	3.1 (1.1–8.9)
Benign tumors	16	25	3.6 (1.8–6.9)	1.9 (0.8–4.4)

*CVID* common variable immunodeficiency*, OR* odds ratio, *95%CI* 95% confidence intervals*, STIs* sexually transmitted diseases*, UTIs* urinary tract infections*, CNS* central nervous system*, IHD* ischemic heart disease*, DM* diabetes mellitus

### Time Trends in Changes in the Relative Risk

We further split the time into three time-periods (< 1, ≥ 1–3, and ≥ 3–5 years) (Table 3). We only analyzed categories that included a minimum of one case and one control and had at least a total of 10 participants. Among the disease categories with the largest association in the five-year period (i.e., lung diseases, LRTI, ENT infections, and gastrointestinal diseases), a major association was observed during all three time periods. Concerning other categories, an association was still observed for all three time periods (i.e. IHD, rheumatological diseases, non-diabetic endocrine diseases, and DM2 and unspecified DM) or one to two periods (gastrointestinal infections, skin infections, sexually transmitted infections (STIs), and other genital infections, kidney and urinary tract infections, sepsis, autoimmune gastrointestinal disorders, autoimmune rheumatic disorders, ENT diseases, eye diseases, neurological diseases, skin diseases, and benign tumors); however, the size of the impact was small. Except for ENT infections and gastrointestinal diseases, no clear increasing trend in risk with shorter time to diagnosis was observed; however, the absolute number of diagnoses during the years was small (Table 3).

**Table 3:** Association between risk of subsequent CVID for 23 disease categories in three time periods prior to index date

Disease category	<1 year OR (95%CI)	≥1–3 years OR (95%CI)	≥3-5 years OR (95%CI)
Infections			
Ear, nose, and throat infections	20.0 (2.2–178.9)	12.5 (2.4–64.4)	5.0 (1.0–24.8)
Lower respiratory tract infections	38.8 (13.7–109.8)	24.8 (7.2–85.5)	32.5 (7.3–144.0)
Gastrointestinal infections	N/A	5.0 (1.0–24.8)	2.5 (0.5–13.7)
Skin infections	5.0 (0.3–79.9)	20.0 (2.2–178.9)	3.3 (0.6–20.0)
STIs and other genital infections	2.5 (0.2–27.6)	1.8 (0.2–20.9)	1.7 (0.2–16.0)
Kidney and UTIs	5.0 (0.3–79.9)	N/A	3.0 (0.7–12.6)
Sepsis	N/A	20.0 (2.2–178.9)	1.7 (0.2–16.0)
Most common autoimmune disorders			
Autoimmune gastrointestinal disorders	12.5 (2.4–64.4)	7.5 (1.3–44.9)	10.0 (0.9–110.3)
Autoimmune rheumatic disorders	N/A	2.5 (0.5–13.6)	4.0 (0.5–29.5)
Autoimmune endocrine disorders	10.0 (1.8–54.6)	3.0 (0.7–12.6)	7.5 (2.1–26.6)
Other disease categories			
Ear, nose, and throat diseases	1.6 (0.3–9.3)	2.9 (0.9–8.5)	2.8 (0.9–9.1)
Eye diseases	2.7 (0.9–8.4)	0.6 (0.1–2.8)	1.7 (0.5–6.5)
Lung diseases	51.8 (15.9–169.1)	27.2 (9.4–78.7)	33.3 (9.9–112.2)
Neurological diseases	2.8 (0.9–8.3)	1.9 (0.7–4.8)	N/A
Skin diseases	5.0 (1.5–17.3)	2.9 (0.8–9.8)	0.8 (0.1–6.9)
Hematological diseases	10.0 (2.5–40.0)	1.7 (0.3–8.3)	7.5 (2.1–26.6)
Gastrointestinal diseases	8.0 (3.8–16.8)	2.7 (1.4–5.1)	2.7 (1.3–5.7)
Cardiovascular and non–IHDs	3.1 (1.6–6.3)	2.6 (1.5–4.8)	4.6 (2.3–9.3)
Ischemic heart diseases	5.0 (1.3–20.0)	3.8 (1.2–12.7)	4.0 (1.1–14.9)
Rheumatological diseases	2.2 (1.1–4.4)	2.0 (1.1–3.6)	1.9 (0.9–3.5)
Non–diabetic endocrine diseases	4.4 (1.8–10.3)	2.3 (1.5–4.8)	3.9 (1.4–10.8)
DM2 and unspecified DM	3.9 (1.5–10.4)	3.6 (1.1–11.3)	7.5 (1.8–32.0)
Benign tumors	7.5 (2.7–21.1)	1.1 (0.2–5.1)	2.0 (0.7–5.3)

*CVID* common variable immunodeficiency*, OR* odds ratio, *95%CI* 95% confidence intervals, *STIs* sexually transmitted diseases, *UTIs* urinary tract infections, *CNS* central nervous system, *IHD* ischemic heart disease, *DM* diabetes mellitus

Only exposures that included a minimum of one case and one control and had at least a total of 10 participants in Table 2 were analyzed

### CVID Indicator Diseases

To narrow down the specific diseases that were associated with a subsequent diagnosis of CVID, we estimated the association with 210 subcategories (for definitions, see Supplementary Information) for the total 5-year period prior to index date (Table 4). We used the same algorithm of analyses and display of our results as explained above. Furthermore, only subcategories for which we observed an at least 3-fold increased OR of subsequent CVID diagnosis are shown in the table (for the remaining results, please view Supplementary Information Table S2). Twenty-eight disease categories showed a statistically significant at least 3-fold increased OR. A major association was found for sixteen diseases. For LRTIs, the strongest association was a diagnosis of other bacterial pneumonia (OR: 60; 95%CI 7.8–461.4); followed by pneumococcal pneumonia (35.0; 4.3–284.5) and unspecified pneumonia (23.2; 10.3–52.4). Other specific diseases in the infectious disease categories that showed an increased risk were acute pharyngitis and sinusitis (20.0; 2.2–178.9), infectious gastroenteritis (8.5; 2.8–25.5), and abscesses/furuncle/carbuncle of the skin (6.7; 1.5–29.8). Concerning a diagnosis for autoimmune disease categories, Graves’ disease (15.0; 1.6–144.2) and type 1 diabetes (5.0; 1.6-15.5) were the only specific diseases with a significant association. Several significant associations were observed in the category “other diseases.” Concerning lung diseases, we found a clear association between chronic obstructive pulmonary disease (COPD) (22.9; 8.7–60.1), asthma (16.9; 6.3–45.8), and other lung diseases (23.6; 5.2–108.2) and a subsequent risk of CVID diagnosis. In the ENT disease category, an association was observed only in the specific category: other diseases in the upper respiratory tract (27.2; 3.3–227.8). Within the eye disease category, disorder of eyelid, lacrimal system, and orbit showed an increased risk (6.7; 1.5–29.8). In hematological diseases, only nutrition deficiency anemia showed an increased risk (5.00; 1.3–20.0). In the gastrointestinal disease category, an association for non-autoimmune gastritis and duodenitis (27.2; 3.3–227.8), non-infective enteritis and colitis (8.2; 2.4–28.0) and other diseases of the intestines and peritoneum (5.6; 2.7–11.7) was observed. Non-autoimmune inflammatory arthropathy was the only disease in the rheumatological diseases category to show a highly significant association (15.0; 1.6–144.2). Among cardiovascular and non-IHD, heart failure (11.7; 3.0–45.1) and arterial and capillary diseases (5.7; 1.5–21.5) showed an increased risk. The following disease categories showed a 3- to 5-fold risk; epilepsy, migraine, and other episodic CNS disorder (neurological diseases), angina pectoris, myocardial infarction and other IHDs (IHD), thyroid diseases and other endocrine disorders (non-diabetic endocrine diseases), type 2 diabetes (DM2 and unspecified DM), and benign tumors related to the skin, joints, and bone (benign tumors) (Table 4).

**Table 4:** Association between risk of subsequent CVID for 210 subcategories in the five years prior to index date

Main disease category	Subcategory	Cases	Controls	OR (95%CI)
Infections				
Ear, nose, and throat infections	Acute pharyngitis and sinusitis	4	<3	20.0 (2.2–179.0)
Lower respiratory tract infections	Pneumococcal pneumonia	7	<3	35.0 (4.3–284.5)
Lower respiratory tract infections	Other bacterial pneumonia	12	<3	60.0 (7.8–461.4)
Lower respiratory tract infections	Unspecified pneumonia	33	8	23.2 (10.3–52.4)
Gastrointestinal infections	Infectious gastroenteritis	9	6	8.5 (2.8–25.5)
Skin infections	Abscesses, furuncle, carbuncle	4	3	6.7 (1.5–29.8)
Most common autoimmune diseases				
Autoimmune endocrine disorders	Graves’ disease	3	<3	15.0 (1.6–144.2)
Autoimmune endocrine disorders	Type 1 diabetes	6	6	5.0 (1.6–15.5)
Other diseases				
Ear, nose, and throat diseases	Other diseases in upper respiratory tract^1^	6	<3	27.2 (3.3–227.8)
Eye diseases	Disorder of eyelid, lacrimal system, and orbit	4	3	6.7 (1.5–29.8)
Lung diseases	COPD	24	7	22.9 (8.7–60.1)
Lung diseases	Asthma	18	7	16.9 (6.3–45.8)
Lung diseases	Other lung diseases^2^	10	3	23.6 (5.2–108.2)
Neurological diseases	Epilepsy, migraine, and other episodic CNS disorder	6	10	3.0 (1.1–8.3)
Hematological diseases	Nutrition deficiency anemia	4	4	5.0 (1.3–20.0)
Gastrointestinal diseases	Non-autoimmune gastritis and duodenitis	6	<3	27.2 (3.3–227.8)
Gastrointestinal diseases	Non-infective enteritis and colitis	7	5	8.2 (2.4–28.0)
Gastrointestinal diseases	Other disease of intestines and peritoneum^3^	17	19	5.6 (2.7–11.7)
Cardiovascular and non-IHDs	Heart failure	7	3	11.7 (3.0––45.1)
Cardiovascular and non-IHDs	Arterial and capillary diseases	5	5	5.7 (1.5–21.5)
Ischemic heart diseases	Angina pectoris	5	7	4.2 (1.2–14.8)
Ischemic heart diseases	Myocardial infarction	3	3	5.0 (1.0–24.8)
Ischemic heart diseases	Other IHDs^4^	5	7	3.8 (1.2–12.7)
Rheumatological diseases	Non–autoimmune inflammatory arthropathy	3	<3	15.0 (1.6–144.2)
Non-diabetic endocrine diseases	Thyroid diseases	8	11	3.8 (1.5–9.7)
Non-diabetic endocrine diseases	Other endocrine disorders^5^	17	31	3.1 (1.6–6.0)
DM2 and unspecified DM	Type 2 diabetes	8	12	3.5 (1.4–8.6)
Benign tumors	Benign tumors related to skin, joints, and bone	4	4	5.0 (1.3–20.0)

*CVID* common variable immunodeficiency, *OR* odds ratio, *95%CI* 95% confidence intervals, *COPD* chronic obstructive pulmonary disease, *CNS* central nervous system, *IHD(s)* ischemic heart disease(s), *DM* diabetes mellitus

^1^Includes allergic rhinitis, nasal polyps, tonsil or adenoid hypertrophy, chronic laryngitis or tracheitis, unspecified diseases in vocal cords or glottis, and other/unspecified diseases in the upper respiratory tract

^2^Includes lung diseases caused by external agents, other interstitial lung disease with fibrosis, other/unspecified diseases in the pleura, other/unspecified diseases in respiratory organs, and diseases in the lungs due to conditions classified elsewhere

^3^Includes disorders in the function of the digestive system after surgery, other/unspecified diseases in the gastrointestinal tract, and diseases of other gastrointestinal organs due to conditions classified elsewhere

^4^Includes coronary thrombosis without infarction, post-myocardial infarction syndrome, and other/unspecified acute IHD

^5^Includes other disorders of sugar regulation and internal secretion of the pancreas, dysfunction of the pituitary gland, Cushing’s syndrome, adrenogenital syndrome, secondary or other/unspecified hyperthyroidism, other diseases of the adrenal gland, disorders in ovary- or testicle-function, unspecified hormonal dysfunction, dysfunction of multiple endocrine glands, diseases in thymus, diseases in other endocrine glands, diseases of other endocrine glands due to conditions classified elsewhere, malnutrition, and other nutrition-related deficiencies

Only exposures that included a minimum of one case and one control and had at least a total of 10 participants in Table 2 were analyzed and only categories with an at least 3-fold increased risk is displayed

## Discussion

In this nationwide, population-based nested case–control study, we found that CVID patients had four times more hospital contact in the five years prior to diagnosis compared to age-, sex-, and region-matched controls from the general population (*p* < 0.001). In the five years prior to the CVID diagnosis, 90.6% of the CVID patients compared to only 42.8% of the controls had at least one hospital contact concerning the 33 major disease categories investigated. Although a diagnosis of infections, mainly LRTIs, and lung diseases were associated with the highest risk (30-fold and 35-fold, respectively) for a subsequent CVID diagnosis, an association was observed concerning a diagnosis for more than 50% of the 33 disease categories and 13% of the 210 subcategories. Importantly, the association was not only observed in the last year before diagnosis, suggesting the possibility for earlier CVID diagnosis.

Previous studies have highlighted infectious and non-infectious diseases present at the CVID diagnoses. However, to our knowledge, this is the first study to identify missed opportunities to diagnose CVID based on the presence of CVID indicator conditions among CVID patients compared to healthy controls in the five years prior to a CVID diagnosis.

A previous study from our group [15] compared CVID patients to age- and sex-matched controls in the three years prior to CVID diagnosis. The study showed that CVID patients had significantly more consultations with their general practitioners (GPs) relative to the controls and showed that approximately 30% of CVID patients had COPD diagnosed at the time of CVID diagnosis. Recent data from our group [22] further showed a clear association between consumption of antibiotics, systemic oral glucocorticoids, and inhaled bronchodilators/glucocorticoids with risk of subsequent CVID diagnosis. These results emphasize the importance of GPs in targeted screening for hypogammaglobulinemia to reduce the diagnostic delay of CVID. In the current study, we focused on hospital admissions and (non-infection-related) visits in the OPD to identify potential indicator conditions that should prompt examinations for CVID regardless of their causal or non-causal relationship with CVID. As anticipated and consistent with previous studies [15, 23, 24], LRTIs and lung diseases demonstrated the highest association with subsequent risk of CVID diagnosis. Although smoking status was unknown, we believe that the occurrence of recurrent pulmonary infections may result in the development of chronic pulmonary diseases and therefore might be a possible complication of the delayed CVID diagnosis. Therefore, our findings highlight the importance of screening for hypogammaglobulinemia in individuals presenting with recurrent/severe respiratory infections and/or lung diseases.

As expected, concerning conditions with a presumed causal link, we found an association with ENT infections, gastrointestinal infections, skin infections, and sepsis and a subsequent CVID diagnosis in the five-year period prior to CVID diagnosis. For autoimmune diseases, which are more prevalent among CVID patients relative to the general population [5, 6], we observed an association between autoimmune gastrointestinal and endocrinological disorders in the five years prior to CVID diagnosis, whereas only insignificant trends were observed for autoimmune rheumatological and dermatological disorders. This is probably due to low prevalence of many of these autoimmune diseases in general and overall low power in our study. Unfortunately, our study design and power of our study failed to give an estimate of autoimmune hematologic disorders, as no controls but 14 cases had an event. Despite this, autoimmune hematologic disorders should indeed be considered an indicator disease as the OR had been almost 80, if only one control had displayed an event. A similar problem exists for spleen disorders where the OR would have been approximately 35 if only one control had displayed an event. In accordance with the results of previous studies on the risk of enteropathy among CVID patients [4, 25], we observed an association between non-autoimmune gastritis and duodenitis, as well as non-infective enteritis and colitis with a subsequent CVID diagnosis. Finally, nutrition deficiency anemia was the only hematological disease to show an increased risk of subsequent CVID, which further emphasizes the malabsorptive conditions observed in CVID patients. It is well-established and recommended in both Danish and European guidelines that clinicians should check for hypogammaglobulinemia in patients presenting with these types of non-infectious diseases [26]. Nevertheless, our results represent missed opportunities in the years leading up to the diagnosis. We are aware that the time from referral until an established CVID diagnosis might take some time. However, no major change in the observed association was found in our sensitivity analysis of year ≥ 1–5 before diagnosis (Table 2) and for many of the major exposures also in the years < 1, ≥ 1–3, and ≥ 3–5 before diagnosis (Table 3), although the absolute number of diagnoses was small in the latter analysis.

In our study, we further examined conditions with no anticipated causal link with CVID. Unexpectedly, many of these conditions were associated with a subsequent risk of CVID. Among these, IHD illustrated a more than 4-fold increased risk. While a direct link between CVID and an increased risk of cardiovascular diseases has not yet been established, the presence of systemic inflammation might increase the risk of cardiovascular events [27]. Furthermore, a more than 3.5-folds increased risk was observed among individuals diagnosed with benign tumors, which was further subclassified to tumors of the skin, joints, and bone. While malignant cancers such as lymphoid or gastric cancers are observed at increased rates among individuals with CVID [4, 6, 13], as well as benign lymphoproliferation [7] and granulomatous processes in several organs [1], we unfortunately did not have access to pathology results or journals, which is why any further exploration of these diagnosis could not be performed. Concerning IHD and benign tumors, and other associations observed in the current study where no causal link has been established (Tables 2 and 3), the associations need to be repeated in other studies to see if these results can be replicated. For the diagnoses with a presumed causal relationship, our data indicates that these conditions could act as indicator conditions which should prompt examination for hypogammaglobulinemia and if established, referral for further investigations. In general, doctors specialized in infectious diseases, pulmonology, gastroenterology, and other relevant clinical fields should be aware of CVID as the most common primary immunodeficiency among adults and aware of the overlap of CVID with diagnosis cared for in their field. However, as CVID is a rare condition, universal testing is probably not a feasible method for improving timely diagnosis. Targeted screening using specific indicator diseases in both primary and secondary care might represent a better solution. Nevertheless, larger studies should assess the numbers needed to test to find one CVID patient based on the results of our analysis.

Another feasible method for earlier diagnosis of CVID could include the use of a computer-based algorithm or scoring system utilizing ICD-10 diagnosis codes for indicator conditions. This method has been utilized in previous studies [28] and is also seen in the medical tool called Software for Primary Immunodeficiency Recognition, Intervention and Tracking (SPIRIT) analyzer, which matches warning signs of primary immunodeficiency with 352 ICD-10 codes to identify at-risk patients [29].

A strength of this study is its use of a nationwide population-based nested cohort consisting of validated CVID patients and age-, gender-, and region-matched controls from the general population. The use of high-quality Danish registries [19, 20] provided us with complete access to information on every individual and data on all diseases diagnosed during the five years prior to CVID diagnosis.

Our study had some limitations. We included nationwide data on CVID patient; however, the rare nature of CVID resulted in a low sample size and low power, especially in our stratified analysis. As a result of this, we were unable to estimate ORs for disease categories where the controls had no events although these disease categories may in fact play an important role as a clinical predictor for CVID. To address problems with multiple testing, we set up certain algorithms for analysis and display of our results. The risk of type 2 errors cannot be excluded; however, the results demonstrate a consistent picture of higher risk for CVID regardless of disease group and timing. To identify the exposure, we relied on hospital registry-based diagnoses. This might have led to some underestimation of the occurrence of certain disease categories that are typically diagnosed in non-hospital settings, thereby potentially underestimated the association with, e.g., ENT infections and ENT diseases. To achieve the most accurate diagnoses, we only used non-emergency room discharge diagnoses and visits to the OPD for non-infectious disease. This might have led to a slight underestimation of the risk of overall infections; however, as previously described our objective was to identify infections leading to admissions. Furthermore, a diagnosis of CVID cannot be made after therapy with immunosuppressive therapy, e.g., rituximab. However, as immunosuppressive therapy is often used for autoimmune conditions without previous testing for hypogammaglobulinemia, we are aware that we might have underestimated the association between autoimmune disease and a subsequent CVID diagnosis. Given this shortcoming, we did not estimate the risk of cancer although we believe lymphoma is a strong indicator. Nevertheless, for these conditions a different study design must be applied. Lastly, we had to rely on hospital registry-based discharge diagnoses in order to identify diagnoses, which may have led to misclassification. However, the positive predictive value of registry diagnoses has previously been shown to be high (70–99%) [30]. Furthermore, we used the same data sources to ascertain the diagnoses for both cases and controls and assume that the accuracy of the data did not vary by CVID status, which minimizes differential misclassification.

In conclusion, this study suggests the potential for earlier CVID diagnosis by using targeted testing for antibody deficiency in patients diagnosed with CVID indicator conditions. Although future studies with larger sample sizes and more comprehensive data are needed to further explore some of these associations, other more well-established associations should be prioritized and actively pursued to reduce the diagnostic delay, thereby leading to earlier treatment and potentially reduced morbidity and mortality.

### Supplementary Information

ESM 1 (PDF 1067 kb)

## Data Availability

The authors confirm that the data supporting the findings of this study are available within the article and/or its supplementary materials.

## References

[1] Bonilla FA, Barlan I, Chapel H, Costa-Carvalho BT, Cunningham-Rundles C, Morena TDL (2016). International consensus document (ICON): common variable immunodeficiency disorders. J Allergy Clin Immunol Pract.

[2] Westh L, Mogensen TH, Dalgaard LS, Bernth Jensen JM, Katzenstein T, Hansen AE (2017). Identification and characterization of a nationwide Danish adult common variable immunodeficiency cohort. Scand J Immunol.

[3] Peng Y, Chen Y, Wang Y, Wang W, Qiao S, Lan J, Wang M. Dysbiosis and primary B-cell immunodeficiencies: current knowledge and future perspective. Immunol Res. 2023;71(4):528–36. 10.1007/s12026-023-09365-5 36933165

[4] Yazdani R, Habibi S, Sharifi L, Azizi G, Abolhassani H, Olbrich P, Aghamohammadi A (2020). Common variable immunodeficiency: epidemiology, pathogenesis, clinical manifestations, diagnosis, classification, and management. J Investig Allergol Clin Immunol.

[5] Mormile I, Punziano A, Riolo CA, Granata F, Williams M, de Paulis A, Spadaro G, Rossi FW (2021). Common variable immunodeficiency and autoimmune diseases: a retrospective study of 95 adult patients in a single tertiary care center. Front Immunol.

[6] Ho HE, Cunningham-Rundles C (2020). Non-infectious complications of common variable immunodeficiency: updated clinical spectrum, sequelae, and insights to pathogenesis. Front Immunol.

[7] Bruns L, Panagiota V, von Hardenberg S, Schmidt G, Adriawan IR, Sogka E (2022). Common variable immunodeficiency-associated cancers: the role of clinical phenotypes, immunological and genetic factors. Front Immunol.

[8] Gathmann B, Mahlaoui N, Gérard L, Oksenhendler E, Warnatz K, Schulze I (2014). Clinical picture and treatment of 2212 patients with common variable immunodeficiency. J Allergy Clin Immunol.

[9] Graziano V, Pecoraro A, Mormile I, Quaremba G, Genovese A (2017). Delay in diagnosis affects the clinical outcome in a cohort of CVID patients with marked reduction of iga serum levels. Clin Immunol.

[10] Slade CA, Bosco JJ, Binh Giang T, Kruse E, Stirling RG, Cameron PU (2018). Delayed diagnosis and complications of predominantly antibody deficiencies in a cohort of Australian Adults. Front Immunol.

[11] Quinti I, Soresina A, Spadaro G, Martino S, Donnanno S, Agostini C (2007). Italian primary immunodeficiency network. Long-term follow-up and outcome of a large cohort of patients with common variable immunodeficiency. J Clin Immunol.

[12] Ziętkiewicz M, Więsik-Szewczyk E, Matyja-Bednarczyk A, Napiórkowska-Baran K, Zdrojewski Z, Jahnz-Różyk K (2020). Shorter diagnostic delay in Polish adult patients with common variable immunodeficiency and symptom onset after 1999. Front Immunol.

[13] Odnoletkova I, Kindle G, Quinti I, Grimbacher B, Knerr V, Gathmann B (2018). The burden of common variable immunodeficiency disorders: a retrospective analysis of the European Society for Immunodeficiency (ESID) registry data. Orphanet J Rare Dis.

[14] Rider NL, Kutac C, Hajjar J, Scalchunes C, Seeborg FO, Boyle M, Orange JS (2017). Health-related quality of life in adult patients with common variable immunodeficiency disorders and impact of treatment. J Clin Immunol.

[15] Ilkjær FV, Rasmussen LD, Martin-Iguacel R, Westh L, Katzenstein TL, Hansen AE (2019). How to identify common variable immunodeficiency patients earlier: general practice patterns. J Clin Immunol.

[16] Janssen LMA, van der Flier M, de Vries E (2021). Lessons learned from the clinical presentation of common variable immunodeficiency disorders: a systematic review and meta-analysis. Front Immunol.

[17] The population in Denmark: https://www.dst.dk/da/Statistik/emner/borgere/befolkning/befolkningstal. Statistics Denmark. Accessed 05 May 2023.

[18] Schmidt M, Schmidt SAJ, Adelborg K, Sundbøll J, Laugesen K, Ehrenstein V (2019). The Danish health care system and epidemiological research: from health care contacts to database records. Clin Epidemiol.

[19] Pedersen CB (2011). The Danish Civil Registration System. Scand J Public Health.

[20] Lynge E, Sandegaard JL, Rebolj M (2011). The Danish National Patient Register. Scand J Public Health.

[21] Søgaard OS, Lohse N, Østergaard L, Kronborg G, Røge B, Gerstoft J (2012). Morbidity and risk of subsequent diagnosis of HIV: a population based case control study identifying indicator diseases for HIV infection. PloS One.

[22] Ilkjær FV, Rasmussen LD, Martin-Iguacel R, Westh L, Katzenstein TL, Nielsen TL, et al. Evaluating of drug prescription patterns in undiagnosed common variable immunodeficiency patients. J Clin Immunol. 2023 (In review). 10.1007/s10875-023-01598-1 37833619

[23] Cabañero-Navalon MD, Garcia-Bustos V, Nuñez-Beltran M, Císcar Fernández P, Mateu L, Solanich X (2022). Current clinical spectrum of common variable immunodeficiency in Spain: the multicentric nationwide GTEM-SEMI-CVID registry. Front Immunol.

[24] Zainaldain H, Rizvi FS, Rafiemanesh H, Alizadeh M, Jamee M, Mohammadi S, Kiaee F (2020). Infectious complications reporting in common variable immunodeficiency: a systematic review and meta-analysis. Oman Med J.

[25] Mohammadi F, Yadegar A, Mardani M, Ayati A, Abolhassani H, Rezaei N (2023). Organ-based clues for diagnosis of inborn errors of immunity: A practical guide for clinicians. Immun Inflamm Dis.

[26] Marquart HV, Heilmann C, Katzenstein T, Larsen CS, Müller K et al: Guidelines for diagnosis and treatment of primary immunodeficiency (2018). The Capital Region of Denmark. Available at: https://www.regionh.dk/blodbanken/afdelingen/enheder-paa-rigshospitalet/Documents/retningslinier-for-diagnostik-og-behandling-af-primaer-immundefekt.pdf. Accessed 23 July 2023.

[27] Fioranelli M, Bottaccioli AG, Bottaccioli F, Bianchi M, Rovesti M, Roccia MG (2018). Stress and inflammation in coronary artery disease: a review psychoneuroendocrineimmunology-based. Front Immunol.

[28] Cunningham-Rundles C, Sidi P, Estrella L, Doucette J (2004). Identifying undiagnosed primary immunodeficiency diseases in minority subjects by using computer sorting of diagnosis codes. J Allergy Clin Immunol.

[29] Modell V, Quinn J, Ginsberg G, Gladue R, Orange J, Modell F (2017). Modeling strategy to identify patients with primary immunodeficiency utilizing risk management and outcome measurement. Immunol Res.

[30] Nickelsen TN (2001). Data validity and coverage in the Danish National Health Registry. A literature review. Ugeskr Laeger.

