# Supplementary material for: Missed Opportunities to Diagnose Common Variable Immunodeficiency: a Population-Based Case–Control Study Identifying Indicator Diseases for Common Variable Immunodeficiency
Source: J Clin Immunol. 2023 Sep 28;43(8):2104–14. doi: 10.1007/s10875-023-01590-9 (PMC10661759; doi:10.1007/s10875-023-01590-9)
Supplement: Supplementary file 1 — (PDF 1067 kb) [file 10875_2023_1590_MOESM1_ESM.pdf]

Title: Missed opportunities to diagnose common variable immunodeficiency: A Population Based Case Control Study Identifying Indicator Diseases for Common Variable Immunodeficiency.

Journal: Journal of Clinical Immunology

Authors: Christina Dahl, Inge Petersen, Frederik V. Ilkjær, Lena Westh, Terese L. Katzenstein, Ann-Brit E. Hansen, Thyge L. Nielsen, Carsten S. Larsen, Isik S. Johansen, Line D. Rasmussen

Corresponding author:
Christina Dahl
Department of Infectious Diseases,
Odense University Hospital
J.B. Winsløwsvej 4,
5000 Odense C
E-mail: ch-cd@hotmail.dk

Appendix, Table S1. *International Classification of Diseases*, version 10 codes used for the analyses.

Disease categories	ICD–10 codes
**INFECTIONS**	
**Ear, nose and throat infections**	H60–H603, H608-H610, H62-H628, H65–H68, H70-H709, H73-H739, H750-H758, H812, H830, J00–J069, J31–J329, J340, J350, J36-J369, J387G, J390-J391
Subcategory	
Infections of external ear	H60–H603, H608-H610, H62-H628
Infections of middle ear and mastoid	H65–H68, H70-H709, H73-H739, H750-H758
Infections of inner ear	H812, H830, H838-H839
Acute pharyngitis and sinusitis	J01–J029
Acute tonsillitis	J03–J039
Other acute upper resp. tract infections	J00–J009, J04–J069
Chronic pharyngitis and sinusitis	J31–J329
Chronic tonsillitis	J350
Abscess in upper resp. tract	J340, J36–J369, J387G, J390-J391
**Eye infections**	B30-B309, H00-H019, H03-H038, H040-H041, H043-H044, H061, H10–H109, H15-H159,

	H16-H160, H162-H169, H19-H198, H20-H209, H22-H228
Subcategory	
Infections of eyelid, lacrimal system, and orbit	H00-H019, H03-H038, H040-H041, H043-H044, H048-H049
Conjunctivitis	B30–B309, H10–H109
Infections of sclera, cornea, iris, and ciliary body	H15-H159, H16-H160, H162-H169, H19-H198, H20-H209, H22-H228
**Lower respiratory tract infections (LRTIs)**	A481, A709, J09–J229, J850-J853
Subcategory	
Bronchitis and non–specific LRTIs	J20–J229
Influenza and viral pneumonia	J09–J129
Pneumococcal pneumonia	J13–J139
Other bacterial pneumonia	A481, A709, J14–J159
Unspecified pneumonia	J16–J189
Lung abscess	J850–J853
**Gastrointestinal infections**	A00-A099, D733, K04-K049, K05-K056, K112-K113, K12-K122, K130A, K140A, K20-K209, K230, K353A-K353B, K570A, K571A, K572A, K578-K579, K61-K614, K630, K65-K659, K67-K678, K75-K751, K77-K778, K810A, K858-K859
Subcategory	
Infections of the teeth	K04-K049, K05-K056
Infection of the salivary glands and oral mucosa	K112-K119, K12-K122
Lip and tung abscess	K130A, K140A
Infectious gastroenteritis	A00-A099
Infections of esophagus	K20-K209, K230

Peritonitis	K65-K659, K67-K678
Infections of liver	K75-K751, K77-K778
Acute cholecystitis	K810
Abscess in the GI canal	D733, K353A-K353B, K570A, K571A, K572A, K578-K579, K61-K614, K630, K650A, K750, K810A, K858A
**CNS infections**	A390, A81–A899, G00–G099, G530-G531
Subcategory	
Bacterial meningitis	A390, G00–G019
Viral meningitis or encephalitis	A81–A899
Other CNS infections	G02–G099, G530-G531
**Skin infections**	A46–A469, B35–B369, L00–L039, L04-L089
Subcategory	
Erysipelas	A46–A469, L032
Pilonidal cyst	L05–L059
Abscess, furuncle, carbuncle	L02–L031, L033–L039
Fungal skin infections	B35–B369
Other skin infections	L00–L019, L04–L089
**Sexually transmitted infections (STIs) and other genital infections**	A50–A649, B20-B249, N30-N399, N41-N419, N45-N459, N481-N482, N49-N499, N512-N518, N70-N735, N738-N739, N75-N764, N77-N778
Subcategory	
Syphilis	A50–A539
Condyloma	A630, A630A
Anogenital herpes simplex	A60–A609
HIV	B20-B249
Other STIs	A54–A599, A61–A629, A631–A649
Infections in male genitalia	N41-N419, N45-N459, N481-N482,

	N49-N499, N512-N518
Infections in female genitalia	N70-N735, N738-N739, N75-N764, N77-N778
**Kidney and urinary tract infections**	N10–N109, N129, N151, N300–N309, N340
Subcategory	
Pyelonephritis	N10–N109, N129
Cystitis	N300–N309
Abscess in urinary tract	N151, N340
Urethritis	N341
**Infections of the heart**	I30–I309, I33–I339, I38–I399, I40–I419
Subcategory	
Peri– and myocarditis	I30–I309, I40–I419
Endocarditis	I33–I339, I38–I399
**Joint and bone infections**	M00–M019, M463-M465, M608A, M868A, M869A
Subcategory	
Septic arthritis	M00–M019
Infectious discitis	M463-M465
Abscess in muscle and bone	M608A, M868A, M869A
**Sepsis**	A40–A419
Subcategory	
Sepsis caused by streptococcus	A40–A409
Sepsis caused by other agents	A41-A419
**Other viral and fungal infections**	A80-A899, A92-A999, B00-B009, B019–B029, B15–B199, B25-B349, B37-B379, B38-B439, B44-B449, B45-B499
Subcategory	
Viral infections in CNS	A80-A899
Viral febrile diseases transmitted by arthropods and viral febrile hemorrhagic diseases	A92-A999
Herpes simplex	B00-B009

Viral infections characterized by skin and mucosa-affections	B01-B099
Herpes zoster	B019–B029
Hepatitis	B15–B199
Cytomegalovirus	B25-B252
Parotitis	B26-B269
Epstein-Barr virus	B27–B279
Unspecified viral illness	B349
Candida infections	B37-B379
Aspergillose	B44-B449
Rare fungal diseases	B38-B439, B45-B499

.

**Other infections**	A15–A199, A20–A389, A391–A459, A47–A480, A482–A499, A65–A708, A71–A809, B50–B579, B58-B589, B60–B999, I891–I891C, K930, N34–N341, N390
Subcategory	
Mycobacterial infections including tuberculosis	A15–A199, A31-A310C, A311–A319, B90–B909
Other bacterial infection, not classified elsewhere	A482-A499
Malaria	B50–B549
Toxoplasmosis	B58-B589
Protozoan infections	B50-B649
Other specified infections	*remaining codes*

MOST COMMON AUTOIMMUNE DISEASES

**Autoimmune hematologic disorders**	D590-D591, D596A, D600, D613, D618-D619, D693, D709, M311A, D86-D869
Subcategory	

Drug-induced autoimmune hematologic anemia	D590
Other autoimmune hematologic anemia	D591
Cold agglutinin disease	D591A
Paroxysmal cold hemoglobinuria	D596A
Chronic aquired pure red cell aplasia	D600
Idiopathic aplastic anemia	D613
Other aplastic anemia	D618
Unspecified aplastic anemia	D619
Idiopathic thrombocytopenic purpura	D693
Unspecified neutropenia	D709
Thrombotic thrombocytopenic purpura	M311A
Sarcoidosis	D86-D869
**Autoimmune gastrointestinal disorders**	K294, K50-K519, K743, K754, K830F, K900
Subcategory	
Chronic atrophic gastritis	K294
Crohns disease	K50-K509
Ulcerative colitis	K51-K519
Primary biliary cirrhosis	K743
Autoimmune hepatitis	K754
Primary sclerosing cholangitis	K830F
Celiac disease	K900
**Autoimmune rheumatic disorders**	M050-M069, M07-M075, M30-M301, M308, M310, M313, M315-M319, M32-M349, M350-M353, M358-M359, M45-M459, M461, M468-M469, M479, M633
Subcategory	
Rheumatoid arthritis	M050-M069
Spondylarthritis (SpA) and psoriatic arthritis (PsA)	M45-M459, M461, M468-M469, M479, M07-M073

ANCA vasculitis and necrotizing vasculitis	M30-M301, M308, M310, M313, M317-M319
Systemic Lupus Erythematosus	M32-M329
Dermatopolymyositis	M33-M339
Systemic sclerosis	M34-M349
Sjogrens syndrome	M350
Behcets disease	M352
Giant cell arteritis	M315-M316
Polymyalgia rheumatica (PMR)	M353
Arthropathy in inflammatory bowel disease	M074-M075
Sarcoid myositits	M633
Other rheumatic autoimmune diseases	M351, M358-M359
**Autoimmune dermatological disorders**	L100-L109, L12-L149, L40-L409, L43-L439, L63-L639, L80-L809
Subcategory	
Psoriasis	L40-L409
Alopecia	L63-L639
Lichen planus	L43-L439
Vitiligo	L80-L809
Other dermatological autoimmune diseases	L100-L109, L12-L149
**Autoimmune endocrine disorders**	E05-E050, E063, E10–E109, E210, E271
Subcategory	
Graves disease	E05-E050
Hashimotos disease	E063
Type 1 diabetes	E10–E109
Addison's disease	E271

OTHER DISEASE CATEGORIES

**Ear, nose and throat diseases**	H604-H607, H611-H619, H69-H699, H71-H729, H738-H749, H758, H80-H811, H813-H829, H831-H839, H90-H959, J30-J309, J33-J339, J341-

	J348, J351-J359, J37-J389, J392-J399
Subcategory	
Diseases of external ear	H604–H607, H611-H619
Diseases of middle ear and mastoid	H69-H699, H71-H729, H738-H749, H758
Diseases of inner ear	H80-H811, H813-H829, H831-H839
Other ear disorders	H90–H959
Other diseases in upper respiratory tract	J30-J309, J33-J339, J341-J348, J351-J359, J37-J389, J392-J399
**Eye diseases**	H02-H029, H042, H045-H060, H062-H063, H11-H138, H161, H17-H189, H21-H219, H25-H279, H281-H289, H30–H359, H361–H599
Subcategory	
Disorder of eyelid, lacrimal system, and orbit	H02-H029, H042, H045-H060, H062-H063,
Disorder of sclera, cornea, iris, and ciliary body	H11-H138, H161, H17-H189, H21-H219,
Disorders of lens	H25–H279, H281–H289
Other eye disease	*remaining codes*
**Lung diseases**	J40–J850, J852–J999
Subcategory	
Chronic bronchitis	J40–J429
COPD	J44–J449
Asthma	J45–J469
Pneumothorax	J93–J939

Lung empyema	J86–J869
Bronchiectasis	J47-J479
Respiratory disease principally affecting the interstitium	J80–839, J842-J849
Other lung disease	*remaining codes*
**Neurological diseases**	G10–G329, G36-G479, G50-G529, G532–G589, G60-G631, G633-G999
Subcategory	
Hereditary, atrophic, extrapyramidal, and degenerative CNS disease	G10–G329
Other demyelinating CNS disease	G36–G372, G374-G379
Epilepsy, migraine and other episodic CNS disorder	G40–G449, G46–G479
Facial nerve disorder	G51–G519
Other nerve, nerve root, plexus disorder	G50–G509, G52–G529, G532-G589
Polyneuropathy	G60-G631, G633-G649
Neuromuscular, muscular, and other neurological diseases	*remaining codes*
**Skin diseases**	L11-L119, L20–L309, L41-L429, L44-L629, L64-L759, L81-L999
Subcategory	
Atopic dermatitis	L20–L209
Seborrhoeic dermatitis	L21, L212–L219
Other dermatitis and eczema	L22–L309
Papulosquamous disorder	L41-L429, L44-L459
Urticaria and erythema	L50–L549
Other skin diseases	*remaining codes*
**Hematological diseases**	D50-D589, D592-D595, D598-D599, D601, D608-D609, D610-D612, D62–D649, D65–D692, D694–D696, D698–D699, D71-D729, D74-D779
Subcategory	
Hemolytic anemia	D55–D589

Nutrition deficiency anemia	D50–D539
Aplastic anemia	D610-D612
Aquired, non-autoimmune hemolytic anemia	D592-D595, D598-D599
Other specified anemia	D601, D608-D609, D62–D648
Coagulation defect, purpura and non–thrombocytopenic hemorrhagic dis.	D65–D692, D698–D699
Thrombocytopenia	D694–D696
Other hematological diseases	*remaining codes*
**Gastrointestinal diseases**	K00-K039, K049, K06–K109, K110-K111, K114-K119, K13–K149, K21–K229, K231-K238, K25-K293, K295-K469, K52-K609, K62-K649, K66-K669, K70–K742, K752-K753, K76-K769, K778, K80-K829, K831-K871, K901-K938
Subcategory	
Disorder of jaws, teeth and supporting structures	K00-K039, K049, K06–K109
Disease of salivary glands, oral mucosa, tongue and lips	K110-K111, K114-K119, K13–K149
Disease of esophagus	K21–K229, K231-K238
Gastric, duodenal, peptic, and gastro–jejunal ulcer	K25–K289
Non-autoimmune gastritis and duodenitis	K29–K293, K295-K299
Dyspepsia	K30–K309

Hernia	K40–K469
Non–infective enteritis and colitis	K52–K529
Fissure of anal and rectal regions	K60–K609
Other disease of intestines and peritoneum	K55–K599, K62–K649, K66-K669
Liver disease	K70–K742, K752-K753, K76-K769, K778
Disorder of gallbladder and biliary tract	K80–K829, K831-K839
Appendicitis	K35–K379
Pancreatitis	K851–K857, K859-K862
Other gastrointestinal disease	*remaining codes*
**Kidney and urinary tract diseases**	N02-N029, N07-N088, N11-N119, N13-N150, N158-N185, N199–N298, N31–N338, N342–N389
Subcategory	
Glomerular disease	N02-N029, N07-N088
Acute, chronic and unspecified renal failure	N17–N185, N199
Urolithiasis	N20–N239
Other kidney diseases	*remaining codes*
**Spleen diseases**	D73-D732, D734-D739, R161
Subcategory	
Abnormality of spleen function	D73-D732
Spleen cyst, infarct and other spleen diseases	D734-D739
Unspecified splenomegaly	R161
**Cardiovascular and non-ischemic heart diseases (non-IHDs)**	I00–I159, I26–I328, I34–I379, I40–I879, I88-I899, I892–I999, G45–G459

Subcategory	
Hypertension	I10–I159
Pulmonary heart disease	I26–I289
Cardiomyopathy	I42–I429
Arrhythmia	I44–I499
Heart failure	I50–I509
Heart valve disease	I34–I379
Ischemic cerebral event	G45–G459, I63–I639
Subarachnoidal/cerebral hemorrhage	I60–I629
Other cerebral vascular diseases	I64–I699
Arterial and capillary diseases	I70–I799
Thrombophlebitis	I80–I809
Varicose veins of lower extremities	I83–I839
Other non–IHD vascular diseases	*remaining codes*
**Ischemic heart disease (IHD)**	I20–I259
Subcategory	
Angina pectoris	I20–I209
Myocardial infarction	I21–I239
Other IHDs	I24–I259
**Rheumatological diseases**	M02-M039, M076-M259, M302-M303, M311-M312, M314, M354-M357, M36-M369, M40-M439, M460-M462, M47-M478, M48-M549, M60-M602, M609-M632, M638-M869, M87-M999
Subcategory	
Non-autoimmune inflammatory arthropathy	M02-M039, M076, M10-M149
Arthrosis	M15–M199
Other joint disorders	M20–M259
Systemic connective tissue disorders	M302-M303, M311-M312,

	M314, M354-M357, M36-M369
Dorsopathy	M40-M439, M460-M462, M47-M478, M48-M549
Disorder of muscle, synovium, tendon	M60-M602, M609-M632, M638-M689
Disorders of bone density and structure	M80–M859
Other rheumatological diseases	*remaining codes*
**Non–diabetic endocrine diseases**	E00–E062, E064-E079, E15–E209, E211-E270, E272-E309, E311-E899
Subcategory	
Thyroid diseases	E00–E049, E051-E062, E064-E079
Obesity	E65–E689
Metabolic disorder	E70–E899
Other endocrine disorder	*remaining codes*
**Diabetes mellitus type 2 (DM2) and unspecified DM**	E11–E149, G590, G632, H280, H360, M142, N083
Subcategory	
Type 2 diabetes	E11–E119
Unspecified DM	E12–E149, G590, G632, H280, H360, M142, N083
**Benign neoplasms**	D10-D369
Subcategory	
Benign tumors related to GI canal	D10-D109, DD12-D139
Benign tumors related to skin, joints and bone	D16-D179, D22-D239
Benign tumors related to genitalia, kidney and urinary tract	D25-D309
Benign tumors related to mammae or other locations in the thorax	D15-D159, D24-D249

Benign tumors related to blood vessels and lymph nodes	D18-D189
Benign tumors related to mesothelial- and connective tissue	D19-D219
Benign tumors related to brain, meninges and other parts of the CNS	D32-D339
Other benign tumors	D11-D119, D14-D149, D31-D319, D36-D369

Appendix, Table S2 Association between risk of subsequent CVID for 210 subcategories in the five years prior to index date

Main disease category	Subcategory	Cases	Controls	OR (95%CI)
**Infections**				
Ear, nose and throat infections	Infections of external ear	<3	<3	5.0 (0.3-80.0)
Ear, nose and throat infections	Acute pharyngtitis and sinusitis	4	<3	20.0 (2.24-179.0)
Lower respiratory tract infections	Pneumococcal pneumonia	7	<3	35.0 (4.32-284.5)
Lower respiratory tract infections	Other bacterial pneumonia	12	<3	60.0 (7.8-461.4)
Lower respiratory tract infections	Unspecified pneumonia	33	8	23.2 (10.3-52.4)
Gastrointestinal infections	Infectious gastroenteritis	9	6	8.5 (2.8-25.5)
Skin infections	Erysipelas	<3	<3	5.0 (0.3-80.0)
Skin infections	Pilonidal cyst	<3	<3	5.0 (0.3-80.0)
Skin infections	Abscesses, furuncle, carbuncle	4	3	6.7 (1.5-29.8)
Skin infections	Other skin infections	<3	<3	5.0 (0.7-35.5)
Kidney and UTIs	Cystitis	4	6	3.6 (0.9-13.6)
Sepsis	Sepsis caused by other agents	3	4	3.8 (0.8-16.8)
**Most common autoimmune diseases**				
Autoimmune gastrointestinal disorders	Ulcerative colitis	<3	3	3.3 (0.6-20.0)
Autoimmune dermatological disorders	Lichen planus	<3	<3	5.0 (0.3-79.9)
Autoimmune endocrine disorders	Type 1 diabetes	6	6	5.0 (1.6-15.5)
Autoimmune endocrine disorders	Primary adrenal insufficiency / Addisons disease	<3	<3	5.0 (0.3-79.9)
**Other diseases**				
Ear, nose, and throat diseases	Diseases of external ear	<3	<3	5.0 (0.3-79.9)
Ear, nose and throat diseases	Other diseases in upper respiratory tract	6	<3	27.2 (3.3-227.8)
Eye diseases	Disorder of eyelid, lacrimal system and orbit	4	3	6.7 (1.5-29.8)
Lung diseases	COPD	24	7	22.9 (8.7-60.1)
Lung diseases	Asthma	18	7	16.9 (6.3-45.8)
Lung diseases	Other lung diseases	10	3	23.6 (5.2-108.2)
Neurological diseases	Hereditary, atrophic, extrapyrimidal and degenerative CNS disease	<3	<3	5.0 (0.3-79.9)
Neurological diseases	Epilepsy, migraine and other episodic CNS disorder	6	10	3.0 (1.1-8.3)
Neurological diseases	Polyneuropathy	<3	<3	5.0 (0.3-79.9)
Skin diseases	Other dermatitis and eczema	<3	3	3.3 (0.6-20.0)
Hematological diseases	Nutrition deficiency anemia	4	4	5.0 (1.3-20.0)
Hematological diseases	Other hematological diseases	<3	<3	5.0 (0.3-79.9)
Gastrointestinal diseases	Disorder of jaws, teeth and supporting structures	3	4	4.3 (0.9-21.9)

Gastrointestinal diseases	Gastritis and duodenitis	6	3	13.7 (2.7-68.5)
Gastrointestinal diseases	Non-infective enteritis and colitis	7	5	8.2 (2.4-28.1)
Gastrointestinal diseases	Dyspepsia	<3	<3	5.0 (0.7-35.5)
Gastrointestinal diseases	Other disease of intestines and peritoneum	17	19	5.6 (2.7-11.7)
Gastrointestinal diseases	Liver disease	<3	<3	5.0 (0.7-35.5)
Gastrointestinal diseases	Appendicitis	<3	3	3.3 (0.6-20.0)
Non-IHD cardiovascular disease	Pulmonary heart disease	<3	<3	10.0 (0.9-110.3)
Non-IHD cardiovascular disease	Heart faliure	7	3	11.7 (3.0-45.1)
Non-IHD cardiovascular disease	Arterial and capillary diseases	5	5	5.7 (1.5-21.5)
Ischemic heart diseases	Angina pectoris	5	7	4.2 (1.2-14.8)
Ischemic heart diseases	Myocardial infarction	3	3	5.0 (1.0-24.8)
Ischemic heart diseases	Other IHDs	5	7	3.8 (1.2-12.7)
Rheumatological diseases	Non-autoimmune inflammatory arthropathy	3	<3	15.0 (1.6-144.2)
Non-diabetic endocrine diseases	Thyroid diseases	8	11	3.8 (1.5-9.7)
Non-diabetic endocrine diseases	Other endocrine disorders	17	31	3.1 (1.7-6.0)
DM2 and unspecified DM	Type 2 diabetes	8	12	3.5 (1.4-8.7)
Benign tumors	Benign tumors related to skin, joints and bone	4	4	5.0 (1.3-20.0)
Benign tumors	Other benign tumors	3	5	3.0 (0.7-12.6)

CVID Common Variable Immunodeficiency, *OR* Odds ratio, *95%C*; 95% confidence intervals, *COPD* Chronic obstructive pulmonary disease, *CNS* Central nervous system, *IHD(s)* Ischemic heart disease(s), *DM* Diabetes mellitus

Only exposures that included a minimum of one case and one control and had at least a total of 10 participants in table 2, were analyzed and only categories with an OR≥3 is displayed.
